# Carcinosarcoma of monoclonal origin arising in a dermoid cyst of ovary: a case report

**DOI:** 10.1186/1471-2407-6-47

**Published:** 2006-03-01

**Authors:** Daniela Cabibi, Anna Martorana, Francesco Cappello, Elisa Barresi, Claudio Di Gangi, Vito Rodolico

**Affiliations:** 1 Istituto di Anatomia Patologica, Facoltà di Medicina, Università degli Studi di Palermo, Italy; 2 Dipartimento di Medicina Sperimentale, Sezione di Anatomia Umana, Università degli Studi di Palermo, Italy; 3 Istituto di Ginecologia ed Ostetricia, Facoltà di Medicina, Università degli Studi di Palermo, Italy

## Abstract

**Background:**

Transformation of a cystic benign teratoma of the ovary into a "carcinosarcoma" has very rarely been reported and its histogenetic origin is still debated.

**Case presentation:**

A case of carcinosarcoma arising from a dermoid cyst is reported. The tumor showed cystic areas delimited by normal squamous epithelium, with transitional areas through dysplastic epithelium to "in situ" and infiltrating squamous cell carcinoma (SCC).

The sarcomatous component showed compact tissue composed of round cells concentrically arranged around small vessels, spindle, and pleomorphic cells with a high nuclear-cytoplasmic ratio.

Positive staining for vimentin, alpha smooth muscle actin and CD10, as well as P53 and P63, was found in the sarcomatous component and in some atypical basal cells of the squamous epithelium, which also showed the usual epithelial markers.

**Conclusion:**

To the best of our knowledge, this is the first case of carcinosarcoma arising from a dermoid cyst in which a histogenetic origin from totipotent stem cells, located in the basal squamous layer, is supported by immunohistochemical findings.

## Background

A dermoid cyst or benign cystic teratoma is one of the most common tumors in women during reproductive life. Malignant transformation within a dermoid cyst is an infrequent occurrence estimated at less than 2% [1]. The most frequent malignant transformations are squamous cell carcinoma (SCC), adenocarcinoma and pure sarcoma. A carcinosarcoma arising in a dermoid cyst is a very rare event, being considered unlikely until a few years ago [2,3]. To date, only three isolated cases have been reported [4-6]. However, two of these were described as a "collision tumor" [5] or "double malignancy" [6], since these tumors showed a well demarcated boundary between the squamous and sarcomatous areas [5], or consisted of a leiomyosarcoma associated with an "in situ squamous cell carcinoma" [6]. In 1996, Arora and Haldane described a neoplasia arising from a dermoid cyst in the ovary, comprising both adenocarcinomatous and fibrosarcomatous areas, and underlined the need to distinguish their case from malignant mixed Mullerian tumours (MMMTs) that have "carcinomatous and sarcomatous components, but are generally solid and lack the characteristic squamous epithelial lined cyst" [4]. We report here a case of carcinosarcoma characterized by the presence of a "squamous epithelial lined cyst" with transitional areas from benign through premalignant to malignant squamous epithelium.

The most widely accepted hypothesis about the histogenesis of carcinosarcoma is that it originates from stem cells, which are present in almost all tissues and may generate different cellular lineages by the multi-step process named "differentiation". The role of stem cells in tumorigenesis has been clearly demonstrated in a number of carcinogenic models [7-9].

In the past, the histogenetic explanation of carcinosarcoma was based on the "multiclonal hypothesis", which proposed an origin from two or more stem cells of distinct mesenchymal and epithelial origins. Recent studies, showing clonal identity among various foci of carcinosarcomas, have supported a "monoclonal hypothesis", which proposes an origin from a single totipotential stem cell that differentiates along distinct epithelial and mesenchymal pathways [10]. Concordance of positive p53 staining in both the epithelial and sarcomatous components of carcinosarcomas has been adduced in support of the monoclonal hypothesis [11].

In this report, we hypothesize that this rare neoplasia originates from totipotent stem cells, located in the basal squamous layer of the dermoid cyst, and we present immunohistochemical evidence of aberrant expression of mesenchymal markers (vimentin and alpha smooth muscle actin), together with positive staining for CD10, P53 and P63, in some atypical basal squamous cells.

## Case presentation

A 69 year-old woman, with abdominal pain, showed a large pelvic mass. She had undergone a colpohysterectomy for uterine descensus without ovariectomy three years earlier. Ultrasound showed an ovarian mass with both solid and cystic areas. The patient underwent a bilateral salpingo-oophorectomy.

The right ovary, surgically resected, was greatly enlarged (27 × 12, 5 × 4 cm). Sections showed cystic areas containing soft yellow material and hairs, as well as solid areas with hemorrhage and necrosis. In contrast, the left ovary appeared normal. Formalin-fixed paraffin-embedded tissues were stained with hematoxilin-eosin and Periodic acid-Schiff reagent. Immunohistochemical studies were performed using the streptavidin-biotin peroxidase method. The antibodies used were against cytokeratins (AE1/AE3, CAM 5.2, MNF 116) (Dako, Denmark), EMA, vimentin, alpha smooth muscle actin, estrogen and progesterone receptors, p53, p63 (all from Biogenex-Menarini, Netherland), CD10 and desmin (both from Diapath-Novocastra, Newcastle, United Kingdom). For each antibody, appropriate positive and negative controls were included.

### Histological findings

**Figure 1:**
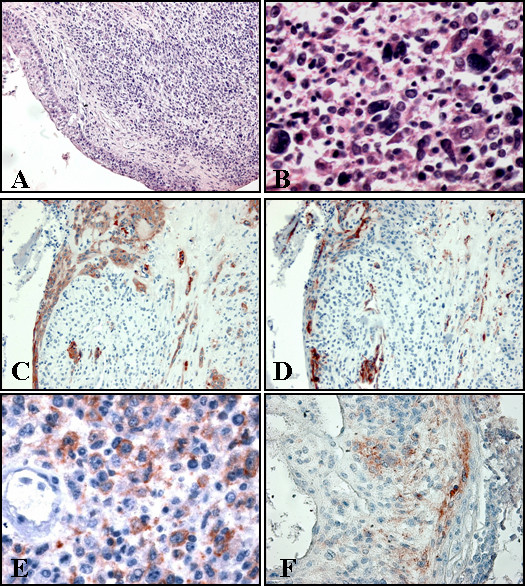
• **A: **Cystic area delimited by benign squamous epithelium, with transitional area to premalignant. Sarcomatous component is present. • **B: **Pleomorphic cells in sarcomatous component. • **C: **Positive staining for CK AE1/AE3 in squamous epithelial lining and squamous infiltrating component. • **D: **Positive staining for alpha smooth muscle actin in basal atypical cells (for control look at positivity in vessels). • **E: **Positive staining for alpha smooth muscle actin in sarcomatous cells. • **F: **Positive staining for CD10 in epithelial basal cells and stromal component.

The tumor had features of a carcinosarcoma with cystic areas delimited by squamous epithelium, focally without atypias (Figure 1a) but with transitional areas from benign through premalignant to "in situ" and infiltrating carcinoma. Hair follicle buds were sometimes present in proximity to the squamous components.

The sarcomatous component showed compact tissue composed of round cells arranged concentrically around small vessels, spindle, and pleomorphic cells with a high nuclear-cytoplasmic ratio (Figure 1b). Necrosis and hemorrhage were abundant. Interestingly, islands of SCC were distributed among sarcomatous areas, without a well-demarcated boundary.

### Immunohistochemical findings

The squamous component stained positively for EMA and cytokeratins AE1/AE3 (Figure 1c) and, MNF 116. In contrast, the stromal component was positive for vimentin, CD10 and, focally, for alpha smooth muscle actin.

Moreover, vimentin, alpha smooth muscle actin and CD10 were positive in atypical basal cells of the squamous epithelium infiltrating both lining cystic areas (Figures 1d–e–f).

Desmin was expressed in fewer than 5% of the stromal cells.

Estrogen receptors were negative. Progesterone receptors were positive in 10% of the sarcomatous cells. P53 and P63 showed positive nuclears staining in about 20% of the tumor cells, in both the sarcomatous and the squamous areas, and in the atypical basal cells of the squamous lining.

## Conclusion

This report describes a case of a partly cystic tumour that showed the "characteristic squamous epithelial lined cyst" described by Arora and Haldane [4]. The epithelial and stromal components were closely intermixed without well-demarcated boundaries, and there were "transitional areas", from the normal, through dysplastic to neoplastic infiltrating squamous epithelium. These features suggest that this neoplasia originated from a dermoid cyst and make the hypothesis of a "collision tumor" less probable.

In our view, it is important to perform a differential diagnosis for an immature teratoma, since Blaustein (1994) hypothesised that on a dermoid cyst "invariably only one tissue element becomes malignant, and the presence of many different malignant elements indicates that the tumor is an immature teratoma and not a mature teratoma that has undergone malignant transformation" [13].

However, an immature malignant teratoma contains elements derived from all three germ layers and, shows a variety of lines of mesenchymal differentiation, notably towards neural elements. Finally it occurs in a younger (pre-menopausal) age group and it is almost never found in women older than 50 years [3,14].

Furthermore, in our opinion, "carcinosarcoma" is the best term for this neoplasia because no immunoreactivity for keratin was detected in the sarcomatous component. On the other hand, "carcinosarcomas and sarcomatoid carcinomas are considered manifestations of the same biologic phenomenon by which the neoplastic cells lose in part or completely their epithelial markers and acquire mesenchymal ones" [15]. This consideration arises from the monoclonal histogenetic theory that is most widely accepted today for the majority of carcinosarcomas [16]. So in accordance with Arora and Haldane [4], we think our case of "carcinosarcoma arising in a dermoid cyst" derives from neoplastic totipotent stem cells with a probabilistic commitment of individual cells and clones to one or other expressed phenotype.

In our case we observed a positive stain for vimentin, alpha smooth muscle actin and CD10 not only in the sarcomatous component, but also in some atypical basal cells of the squamous epithelium.

**Figure 2:**
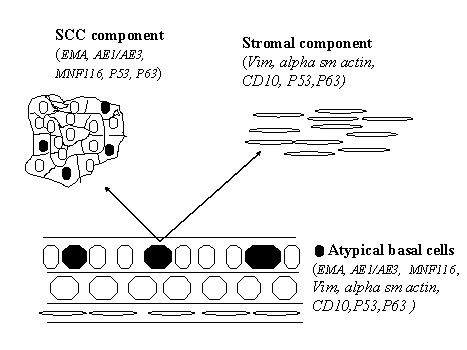
The "divergent monoclonal origin of carcinosarcoma", modified from Thompson E. et al. (15). See the text for further details.

The aberrant expression of these markers, unusual in normal squamous basal cells, suggests that they represent totipotent stem cells that are capable of commitment towards both epithelial and mesenchymal differentiation (Figure 2), and is difficult to reconcile with the hypothesis that they originate from the proliferation of two distinct clones, committed respectively to epithelial and connective differentiation.

Moreover, like Mayall et al. [15], we found concordant positive staining for p53 and p63 in both the epithelial and sarcomatous components, and, notably in our view, in the atypical basal cells of the squamous lining.

We think this case is interesting beceuse a carcinosarcoma-sarcomatoid carcinoma rarely arises from a dermoid cyst and because, to our knowledge, this is the first literature report of immunohistochemical findings that support the hypothesis that a carcinosarcoma can arise from neoplastic totipotent stem cells located in the basal layer of the squamous epithelium.

## Competing interest

The author(s) declare that they have not competing interests.

## Authors' contributions

DC designed the study and drafted the manuscript.

AM collected the specimens and carried out the immunohistochemistry.

FC participated in the draft of the manuscript.

EB participated in the examination of the immunohistochemistry.

CDG participated in the collection of specimens.

VR participated in the design of the study and in the draft of the manuscript

## Pre-publication history

The pre-publication history for this paper can be accessed here:



## References

[B1] Pantoja E, Rodriguez-Ibanez I, Axtmayer RW, Noy MA, Pelegrina I (1975). Complications of dermoid tumors of the ovary. Obstet Gynecol.

[B2] Russel P, Bannatyne P (1989). Surgical pathology of the ovaries. Churchill Livingstone.

[B3] Rosai J, Mosby (1996). Female reproductive system. Ackerman's: Surgical pathology.

[B4] Arora DS, Haldane S (1996). Carcinosarcoma arising in a dermoid cyst of the ovary. J Clin Pathol.

[B5] Hanada M, Tsujimura T, Shimizu H (1981). Multiple malignancies (squamous cell carcinoma and sarcoma) arising in a dermoid cyst of the ovary. Acta pathol Jpn.

[B6] Tyagi SP, Maheshwari V, Tyagi N, Tewari K (1993). Double malignancy in a benign cystic teratoma of the ovary. Indian J Cancer.

[B7] Pathak S (2002). Organ- and tissue-specific stem cells and carcinogenesis. Anticancer Res.

[B8] Pomara G, Cappello F, Cuttano MG, Rappa F, Morelli G, Mancini P, Selli C (2004). Primitive Neuroectodermal Tumor (PNET) of the kidney: a case report. BMC Cancer.

[B9] Sell S, Pierce GB (1994). Maturation arrest of stem cell differentiation in a common pathway for the cellular origin of teratocarcinomas and epithelial cancers. Lab Invest.

[B10] Thompson L, Chang B, Barsky SH (1996). Monoclonal origins of malignant mixed tumors (carcinosarcomas). Evidence for a divergent histogenesis. Am J Surg Pathol.

[B11] Mayall F, Rutty K, Campbell F, Goddard H (1994). P53 immunostaining suggest that the uterine carcinosarcomas are monoclonal. Histopathology.

[B12] Silverberg SG, Major FJ, Blessing JA, Fetter B, Askin FB, Liao SY, Miller A (1990). Carcinosarcoma (malignant mixed mesodermal tumor) of the uterus. A Gynecologic Oncologic Group pathologic study of 203 cases. Int J Gynecol Pathol.

[B13] Kurman RJ, Springer-Verlag (1994). Surface epithelial-stromal tumors of the ovary. Balustein's: Pathology of the femal genital tract.

[B14] Mills SE, Carter D, Greenson J, Oberman HA, Reuter V, Stoler MH (2004). Ovarian surface epithelial stromal tumors. Sternberg's Diagnostic Surgical Pathology.

[B15] Rosai J (2004). Respiratory tract. Ackerman's: Surgical pathology.

[B16] WG McCluggage (2002). Malignant biphasic uterine tumours: carcinosarcomas or metaplastic carcinomas?. J Clin Pathol.

